# Thermal Acclimation and Adaptation in Marine Protozooplankton and Mixoplankton

**DOI:** 10.3389/fmicb.2022.832810

**Published:** 2022-03-23

**Authors:** Albert Calbet, Enric Saiz

**Affiliations:** Institut de Ciències del Mar, CSIC, Barcelona, Spain

**Keywords:** microzooplankton, protist, mixotroph, temperature, grazing, growth, climate change, adaptation

## Abstract

Proper thermal adaptation is key to understanding how species respond to long-term changes in temperature. However, this is seldom considered in protozooplankton and mixoplankton experiments. In this work, we studied how two heterotrophic dinoflagellates (*Gyrodinium dominans* and *Oxyrrhis marina*), one heterotrophic ciliate (*Strombidium arenicola*), and one mixotrophic dinoflagellate (*Karlodinium armiger*) responded to warming. To do so, we compared strains adapted at 16, 19, and 22°C and those adapted at 16°C and exposed for 3 days to temperature increases of 3 and 6°C (acclimated treatments). Neither their carbon, nitrogen or phosphorus (CNP) contents nor their corresponding elemental ratios showed straightforward changes with temperature, except for a modest increase in P contents with temperature in some grazers. In general, the performance of both acclimated and adapted grazers increased from 16 to 19°C and then dropped at 22°C, with a few exceptions. Therefore, our organisms followed the “hotter is better” hypothesis for a temperature rise of 3°C; an increase of >6°C, however, resulted in variable outcomes. Despite the disparity in responses among species and physiological rates, 19°C-adapted organisms, in general, performed better than acclimated-only (16°C-adapted organisms incubated at +3°C). However, at 22°C, most species were at the limit of their metabolic equilibrium and were unable to fully adapt. Nevertheless, adaptation to higher temperatures allowed strains to maintain physiological activities when exposed to sudden increases in temperature (up to 25°C). In summary, adaptation to temperature seems to confer a selective advantage to protistan grazers within a narrow range (i.e., ca. 3°C). Adaptation to much higher increases of temperatures (i.e., +6°C) does not confer any clear physiological advantage (with few exceptions; e.g., the mixotroph *K. armiger*), at least within the time frame of our experiments.

## Introduction

The progressive increase of temperature due to anthropogenic sources (Intergovernmental Panel on Climate Change [IPCC], 2014) will certainly affect planktonic communities in the coming future. This is because temperature is a major factor driving biological activity; the resulting changes in the fitness of a species in response to temperature may provide a selective advantage/disadvantage in comparison to other coexisting species (Halsband-Lenk et al., 2002). This rule applies to all marine organisms, including protistan grazers, such as microzooplankton (pure heterotrophic protists) and mixoplankton (autotrophic protists with phagotrophic capability). Both groups of protistan grazers, which encompass many ciliates and dinoflagellates, are key components of marine pelagic food webs because of their functions as major grazers of phytoplanktonic primary production and as very important prey for larger zooplankton (Calbet, 2001; Calbet and Saiz, 2005; Flynn et al., 2019; Traboni et al., 2021).

Despite the relevance of temperature, most ecophysiological studies addressing its direct effects on protistan grazers do not include multigenerational effects and typically only address short-term responses to variations in environmental temperature [see review by Montagnes et al. (2003)]. Therefore, the actual response of protist grazers to gradual and longer-term temperature changes in marine systems remains essentially unexplored. Conversely, long-term adaptations to temperature have been studied in planktonic algae, and the results have been very revealing and occasionally opposite to those that were expected. For instance, the accepted faster thermal response of respiration vs. photosynthesis, predicted by the metabolic theory of ecology (López-Urrutia et al., 2006; Rose and Caron, 2007), may not be the same after proper adaptation (>100 generations) to environmental conditions (Padfield et al., 2016; Barton et al., 2020). This is because basal metabolism diminishes after genetic adaptation to temperature (Padfield et al., 2016; Barton et al., 2020). Other observed changes in temperature-adapted species are stoichiometric (lower C:N ratios) and related to more efficient use of carbon (Padfield et al., 2016; Aranguren-Gassis et al., 2019). The elemental composition of an organisms is the result of the balance between its metabolic demands and the relative supply of elements in the environment (Sterner and Elser, 2002). It is assumed that phytoplankton exhibit wide variations in their elemental composition and protozoans are more homoeostatic, showing a narrower range of variation (Sterner and Elser, 2002; Klausmeier et al., 2004). However, several studies have shown that the elemental stoichiometry of protozoans may vary significantly in response to the environment and prey composition (Hantzsche and Boersma, 2010; Malzahn et al., 2010; Meunier et al., 2012), and may affect the energy transfer to upper trophic levels (Traboni et al., 2021). The incorporation of elemental ratios and absolute elemental contents (dependent on cellular volume) of different planktonic groups into ecosystem models could improve our ability to predict the response of planktonic communities to environmental threats, and help to understand their influence on the biogeochemistry of the ocean (Litchman et al., 2013; Meunier et al., 2017). Little is known, however, about protistan grazers, both mixotrophic and heterotrophic, in this respect.

We hypothesize that because of the different activation energies between physiological processes, a rise in temperature will favor phagotrophy over photosynthetic carbon acquisition in mixotrophs (Wilken et al., 2013; Lin et al., 2018). We could also hypothesize that following the von Bertalanffy-Perrin model, which states that catabolism is more affected by temperature than anabolism (Perrin, 1995; Li et al., 2011), higher temperatures will enhance respiratory losses to a larger extent than ingestion in protozooplankton (at least for short-term responses). This imbalance should result in a reduction of gross growth efficiency (GGE; Straile, 1997; Li et al., 2011) and perhaps in lower cellular C:N and C:P ratios, depending on the temperature-sensitivity of the excretion response (Alcaraz et al., 2013). We cannot discard, however, that fully temperature-adapted species may not show such differences, as previously reported for algae (Padfield et al., 2016; Barton et al., 2020). This hypothesis, if true, could have very relevant consequences for understanding the functioning of the marine food web and for biochemical modeling ecosystems.

In this study, we aim to explore the responses (volume, stoichiometry growth, and grazing) of different protistan grazers to temperature rise after a short-term exposure (acclimation) and compare them to the performances of long-term adapted organisms. By working with temperature-adapted species, we will also be able to test whether protistan grazers follow the “hotter is better hypothesis” (Bennett, 1987), which predicts that organisms adapted to lower temperatures will have lower maximum performances than those adapted to higher temperatures (Knies et al., 2009). Finally, we also aim to investigate the response of the strains adapted to different temperatures to an extreme temperature episode, here represented by a sudden increase in temperature to 25°C. Heatwaves may become a relevant instrument of species selection in a future scenario of a warmer ocean, where many species will likely be at the edge of their physiological limits. The reported increases of temperature during heatwaves do not reach >6.5°C (Hobday et al., 2016). However, we wanted to go one step further and expose the experimental organisms to relatively extremer temperatures (up to +9°C), to evaluate their survival and performance under critical conditions, and how temperature-adaptation would modify this response. Overall, understanding the processes involved in the thermal adaptation of protozooplankton and mixoplankton and their response to warming will improve the accuracy of climate change models in predicting ecological or biogeochemical effects of temperature projections in the near future.

## Materials and Methods

### Experimental Organisms

For the experiments, we used cultures of two heterotrophic dinoflagellates (*Gyrodinium dominans* and *Oxyrrhis marina*), one heterotrophic ciliate (*Strombidium arenicola*), and one mixotrophic dinoflagellate (*Karlodinium armiger*), all of which originated from the NW Mediterranean. *G. dominans* (ICM-ZOO-GD001) was isolated in February 2011, then kept at 19°C, and transferred to 16°C in June 2019 and to 22°C in November 2020. *O. marina* (ICM-ZOO-OM001) was isolated in 1995 and kept at 19°C; then, it was transferred to 16°C in June 2019 and from 19 to 22°C in November 2020. *S. arenicola* (ICM-ZOO-SA001) was isolated in April 2017, kept at 19°C, and transferred to 16°C in July 2020 and from 19 to 22°C in November 2020. Finally, *K. armiger* (ICM-ZOO-KA001) was isolated in winter 2013, kept at 19°C, and transferred to 16°C in June 2019 and from 19 to 22°C in November 2020. The cultures were kept in temperature-controlled incubators in 260 mL untreated tissue culture PTE flasks with autoclaved 0.1-μm filtered seawater, at a salinity of 38, ca. 35 μmol photons m^–2^ s^–1^, and they were fed exponentially growing *Rhodomonas salina* (K-0294) every 1–2 weeks. We made sure that all the species were kept at the selected temperatures for at least 7 months before conducting the experiments.

### Elemental Analysis

We prepared stock cultures of the adapted strains of all species and starved them. Once no prey was detected both visually and with the aid of a Multisizer IV Coulter Counter, we waited for an additional 24 h to ensure digestion of any remaining prey in the food vacuoles. To evaluate the changes in the stoichiometry of prey during the incubations, we also incubated cultures of *R. salina* (grown at 19°C) at 16, 19, 22, and 25°C for 24 and 48 h. Then, we filtered aliquots of known grazer and prey concentrations onto pre-combusted (450°C, 5 h) GF/F filters (Whatman, 25 mm) for determination of the carbon (C), nitrogen (N), and phosphorus (P) elemental compositions. The filters for CN analysis were dried at 60°C for 48–72 h and then stored in a desiccator until processing with a Flash EA1112 (Thermo Finnigan, München, Germany) CHNS analyzer. The filters for P analysis were immediately frozen at −80°C until processing. We processed P samples with the acid persulfate digestion method and posterior conversion to dissolved inorganic P with a Seal Analytical AA3 (Bran + Luebbe) analyzer. We calculated stoichiometric ratios as molar ratios, and we considered error propagation (square root law) in the calculation of the CP and NP ratios (Saiz et al., 2020).

### Acclimation vs. Adaptation Responses Experimental Setup

We compared strains grown for multiple generations (>7 months) at 16, 19, and 22°C (adapted treatments) with those from 16°C exposed for a short period (2 days preconditioning and 1 day experiment) to a temperature rise of either 3°C (19°C) or 6°C (22°C) (acclimated treatments). These temperatures are within the range of annual oscillation in the area of study and isolation of the microbial grazers used (Calbet et al., 2001). The protocol used was the same for all species and temperatures; i.e., adapted strains incubated at their respective long-term temperature and those incubated at a different temperature followed the same 2d + 1d protocol. The 2-days acclimation took place in 620 mL Pyrex glass bottles submerged in a temperature-controlled bath at the previously noted temperatures. Light conditions were a 10:14 light/dark cycle at 15–20 μmol photons m^–2^ s^–1^. We used saturating prey concentrations for the experiments. Thus, *G. dominans* and *K. armiger* were incubated with 50,000 *R. salina* mL^–1^ (Calbet et al., 2013; Martínez and Calbet, unpublished), whereas *O. marina* and *S. arenicola* were supplied with 100,000–120,000 *R. salina* mL^–1^ (Calbet et al., 2013; Arias and Calbet, unpublished). We added 20 mL of f/2 medium per liter of suspension in all treatments to avoid nutrient deficiency. After the 2 days of acclimation grazers usually had grown and prey were partially depleted. Therefore, we readjusted the prey and grazer concentrations to the ones indicated before, added nutrients again (20 mL f/2 L^–1^), and sequentially transferred the organisms to triplicate 75 mL untreated tissue culture flasks (Falcon), where they were incubated for another 24 h (experiment). The grazer concentrations were chosen to allow a decrease in prey between 10–20%. We also set triplicate control bottles with only *R. salina* at each temperature. Initial and final samples were quantified using a Multisizer IV Coulter Counter, which provided data on abundance and cell volume (Multisizer™ user’s manual, 2010). Predator growth rates were calculated in cell numbers assuming exponential growth. To obtain grazing rates and average prey concentrations during the incubations, we used Frost’s (1972) equations; we calculated per capita values using the average concentration of grazers in each replicate according to Heinbokel (1978). GGEs were calculated as the quotient between carbon-based specific growth and ingestion rates x 100.

### Extreme Thermal Exposure Experimental Setup

We used the same experimental setup described before. However, here, we exposed all the temperature-adapted strains to 25°C for 3 days (two pre-conditioning and one experiment). This temperature is around the maximum average summer temperatures experienced in the area of origin of the species (Calbet et al., 2001). Then, we compared the growth, grazing, and GGEs of the adapted strains at their normal temperature with those after exposure to 25°C. Similar to the previous experiment, we used Frost’s (1972) equations to obtain ingestion rates.

### Statistical Analysis

We used GraphPad Prism 7.0 software to conduct the statistical analysis. For stoichiometric effects of temperature, we sought for significant linear responses in the relationship between temperature and elemental composition or elemental ratio. Regarding experiments, our main objective was to investigate whether the physiological response of temperature-adapted organisms was different from that of only acclimated ones. To do such comparisons, we conducted ANOVA tests, with Tukey’s test to compare the treatments at each temperature, and assuming a *p* < 0.05 for significant differences. Our experimental design rendered more information, such as the different response of both adapted and acclimated organisms to temperature; the effects of temperature on each physiological activity were evaluated by ANOVA, with Tukey’s test to compare the response of each temperature. The differences in the slope of the relationship between temperature (16–19°C) and growth rates (d^–1^) for the different species were evaluated with ANCOVA tests (see Section Acclimation vs. Adaptation). Error propagation, when necessary, was calculated using the square root law (Saiz et al., 2020).

## Results

### Elemental Composition

Table 1 presents the elemental compositions of the temperature-adapted predators. No statistically significant relationship between temperature and either elemental composition or elemental molar ratios was observed in any of the grazers (simple linear regression analysis, *P* > 0.05), with only two exceptions. The P contents of *K. armiger* slightly increased with temperature (from 0.0030 to 0.0039 pgP μm^–3^), and the C:P ratio of *O. marina* diminished with temperature to some degree (from 71 to 64). Table 2 presents the volume and elemental composition of *R. salina* after exposure for 24 and 48 h to the experimental temperatures. This information is relevant for detecting any change in the nutritional composition that the temperature may have produced during the incubations. The volume and CNP composition of *R. salina* were relatively stable and unaffected by exposure at the different temperatures within the incubation periods (variations always <20% with respect to 16°C, and no statistically significant pattern with temperature).

**TABLE 1:** Elemental contents and molar stoichiometric ratios of the temperature-adapted grazers.

Species	Adaptation T (°C)	pgC μm^–3^	SE	pgN μm^–3^	SE	pgP μm^–3^	SE	C:N	SE	C:P	SE	N:P	SE
*G. dominans*	16	0.11	0.005	0.017	0.001	0.0026	0.00016	7.7	0.05	113	2.0	14.7	0.33
*G. dominans*	19	0.09	0.001	0.016	0.0002	0.0028	0.00001	7.1	0.08	86	0.6	12.2	0.18
*G. dominans*	22	0.12	0.003	0.020	0.001	0.0032	0.00002	7.0	0.14	96	2.6	13.7	0.61
*O. marina*	16	0.11	0.003	0.025	0.0007	0.0040	0.0001	5.2	0.03	**71.0**	3.2	13.7	0.70
*O. marina*	19	0.11	0.004	0.025	0.0007	0.0044	0.0002	5.3	0.07	**67.3**	1.5	12.8	0.37
*O. marina*	22	0.10	0.001	0.023	0.0005	0.0040	0.0001	5.1	0.04	**64.4**	2.0	12.7	0.48
*S. arenicola*	16	n.d.	n.d.	n.d.	n.d.	n.d.	n.d.	n.d.	n.d.	n.d.	n.d.	n.d.	n.d.
*S. arenicola*	19	0.09	0.003	0.019	0.0008	0.0034	0.00007	5.2	0.09	67.0	2.2	12.8	0.46
*S. arenicola*	22	0.10	0.003	0.024	0.0008	0.0038	0.00007	5.0	0.02	70.0	2.3	14.0	0.49
*K. armiger*	16	0.21	0.005	0.033	0.0007	**0.0030**	0.00002	7.3	0.01	178	5.3	24.2	0.72
*K. armiger*	19	0.13	0.004	0.027	0.0008	**0.0034**	0.00004	5.7	0.10	99	1.8	17.3	0.44
*K. armiger*	22	0.18	0.004	0.026	0.0004	**0.0039**	0.00001	8.2	0.08	121	2.8	14.7	0.23

*Statistically significant linear relationships with temperature are indicated in bold. n.d. not determined because loss of samples.*

**TABLE 2:** Cell volume, elemental contents and molar stoichiometric ratios of *Rhodomonas salina* after 24 and 48 of exposure to the experimental temperatures.

Exposure time	T	Volume (μm^3^)	SE	pgC μm^–3^	SE	pgN μm^–3^	SE	pgP μm^–3^	SE	C:N	SE	C:P	SE	N:P	SE
24 h	16	208	0.51	0.18	0.002	0.039	0.0006	0.0036	0.00002	5.4	0.02	128	2.3	23.5	0.49
24 h	19	198	0.26	0.17	0.008	0.036	0.0020	0.0036	0.00004	5.5	0.04	120	5.1	21.6	1.06
24 h	22	202	0.49	0.17	0.003	0.037	0.0009	0.0041	0.00015	5.4	0.02	108	3.8	20.0	0.68
24 h	25	209	0.66	0.16	0.001	0.034	0.0003	0.0028	0.00000	5.7	0.02	153	1.0	27.0	0.23
48 h	16	211	0.94	0.18	0.004	0.039	0.0011	0.0040	0.00003	5.4	0.04	117	3.3	21.8	0.79
48 h	19	202	0.52	0.18	0.010	0.038	0.0029	0.0038	0.00003	5.5	0.11	118	6.0	21.7	1.51
48 h	22	207	2.00	0.17	0.007	0.036	0.0016	0.0039	0.00004	5.5	0.04	115	4.9	20.9	1.03
48 h	25	212	1.66	0.20	0.004	0.042	0.0012	0.0050	0.00001	5.7	0.09	105	2.3	18.5	0.51

*No significant relationship was found between temperature and any of the variables analyzed.*

### Acclimation vs. Adaptation

**FIGURE 1:**
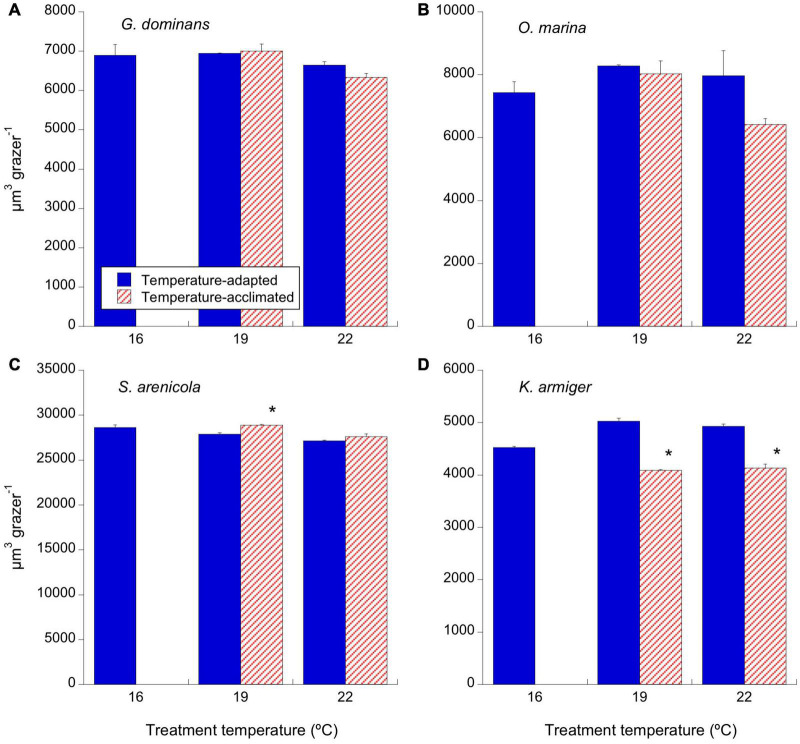
Effects of temperature on cell volume (μm^3^) of the different grazers. Solid blue bars correspond to the temperature-adapted organisms incubated at their standard temperature. Stripped red bars correspond to those organisms adapted at 16°C and exposed to either +3°C (19°C) or +6°C (22°C) temperature raise (2 days acclimation plus 1-day experimental incubation). **(A)** *G. dominans*, **(B)** *O. marina*, **(C)** *S. arenicola*, and **(D)** *K. armiger*. Asterisks denote differences (*p* < 0.05) between acclimated and adapted treatments. The statistics corresponding to differences between temperature treatments of adapted and acclimated organisms can be found in Table 3. The error bars are SE.

Our goal was to compare the physiologies and behaviors of the thermal-adapted strains with those of the parental 16°C strains when exposed to warmer temperatures (acclimated treatment). The focus was on cell volume, growth rates (d^–1^), ingestion rates (in cells ind^–1^ d^–1^), and GGE. For simplicity, we present the significance of the comparison between temperature-adapted strains with their acclimated counterparts in the figures (asterisks indicating *p* < 0.05), whereas the significance of the differences between the response to the different temperatures for adapted and acclimated strains is shown in Table 3. Compared to the 16°C strain, *G. dominans* and *O. marina* cell volumes slightly (although not significantly) augmented with a 3°C temperature increase (19°C), and warmer conditions (+6°C) resulted in a decrease in volume (not statistically significant either), both in adapted and acclimated strains (Figures 1A,B and Table 3). The effect of temperature on *S. arenicola* cell volume was weak for both acclimated and adapted strains (Figure 1C and Table 3). The treatments of 16°C-adapted *S. arenicola* incubated at +3°C (19°C-acclimated treatment) was the only one significantly different from its adapted counterpart (Figure 1C). Finally, the acclimated strains of *K. armiger* decreased in volume at higher temperatures (19 and 22°C) compared to the adapted counterparts (Figure 1D and Table 3). Among the adapted strains of this species, the ones at warmer temperatures showed higher cell volumes than those at 16°C (Table 3), and their volumes were larger than their temperature-acclimated counterparts (Figure 1D; *p* < 0.001).

**TABLE 3:** Probability values of the ANOVA tests comparing the response to temperature in Volume (μm^3^), Growth rates (d^–1^), Ingestion rates (cells grazer^–1^ d^–1^), and GGE (gross-growth efficiency) of the four species of protistan grazers studied.

	*G. dominans*				*O. marina*			
Treatment	Volume	Growth rates	Ingestion rates	GGE	Volume	Growth rates	Ingestion rates	GGE
ANOVA temperature-acclimated	ns	0.002	<0.0001	<0.0001	0.034	0.016	0.0004	ns
Adapted to 16°C vs. Acclimated to +3°C	ns	ns	0.0003	0.007	ns	0.028	0.002	ns
Adapted to 16°C vs. Acclimated to +6°C	ns	0.003	<0.0001	<0.0001	ns	0.021	0.0004	ns
Acclimated to +3°C vs. Acclimated to +6°C	ns	0.007	0.0002	0.002	0.030	ns	ns	ns
ANOVA temperature-adapted	ns	0.009	<0.0001	0.0002	ns	<0.0001	<0.0001	ns
Adapted to 16°C vs. Adapted to 19°C	ns	0.010	<0.0001	0.004	ns	<0.0001	<0.0001	ns
Adapted to 16°C vs. Adapted to 22°C	ns	0.026	<0.0001	0.0001	ns	0.026	0.003	ns
Adapted to 19°C vs. Adapted to 22°C	ns	ns	ns	0.0078	ns	0.0006	0.0004	ns

	***S. arenicola***				***K. armiger***			
	**Volume**	**Growth rates**	**Ingestion rates**	**GGE**	**Volume**	**Growth rates**	**Ingestion rates**	**GGE**

ANOVA temperature-acclimated	0.007	0.0002	<0.0001	ns	0.001	0.004	0.001	0.001
Adapted to 16°C vs. Acclimated to +3°C	ns	0.0002	<0.0001	ns	0.001	0.017	0.002	0.001
Adapted to 16°C vs. Acclimated to +6°C	0.006	0.001	<0.0001	ns	0.002	ns	0.001	ns
Acclimated to +3°C vs. Acclimated to +6°C	ns	0.108	ns	ns	ns	0.004	ns	0.002
ANOVA temperature-adapted	0.029	<0.0001	<0.0001	ns	0.001	0.009	0.002	ns
Adapted to 16°C vs. Adapted to 19°C	ns	<0.0001	<0.0001	ns	0.001	0.011	0.006	ns
Adapted to 16°C vs. Adapted to 22°C	ns	0.0002	<0.0001	ns	0.002	0.022	0.003	ns
Adapted to 19°C vs. Adapted to 22°C	0.030	<0.0001	<0.0001	ns	ns	ns	ns	ns

**FIGURE 2:**
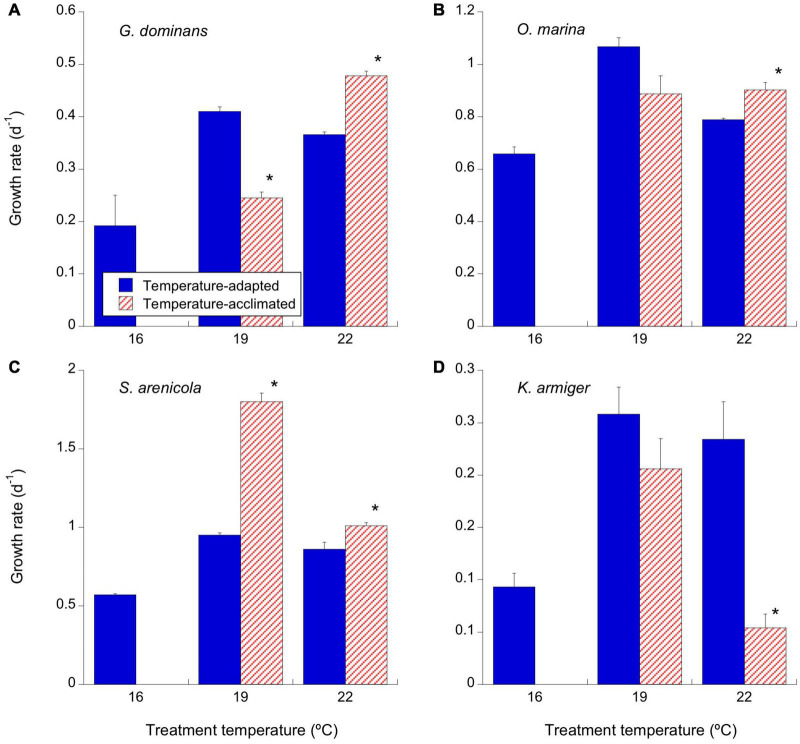
Effects of temperature on the growth rates (d^–1^) of the experimental protistan grazers. Solid blue bars correspond to the temperature-adapted organisms incubated at their standard temperature. Stripped red bars correspond to those organisms adapted at 16°C and exposed to either +3°C (19°C) or +6°C (22°C) temperature raise (2 days acclimation plus 1-day experimental incubation). **(A)** *G. dominans*, **(B)** *O. marina*, **(C)** *S. arenicola*, and **(D)** *K. armiger*. Asterisks denote differences (*p* < 0.05) between acclimated and adapted treatments. The statistics corresponding to differences between temperature treatments of adapted and acclimated organisms can be found in Table 3. The error bars are SE.

*For each comparison, we provide first the overall probability of the ANOVA test and next the posteriori Tukey’s test for each combination of temperature responses. Temperature acclimated corresponds to the results of organisms adapted to 16°C and then incubated at +3 and +6°C. Temperature adapted correspond to the comparison of the performance of each species incubated at the temperature they have been cultivated for multiple generations. The actual data appears in Figures 1, 2, 4, 5. ns = not significant.*

**FIGURE 3:**
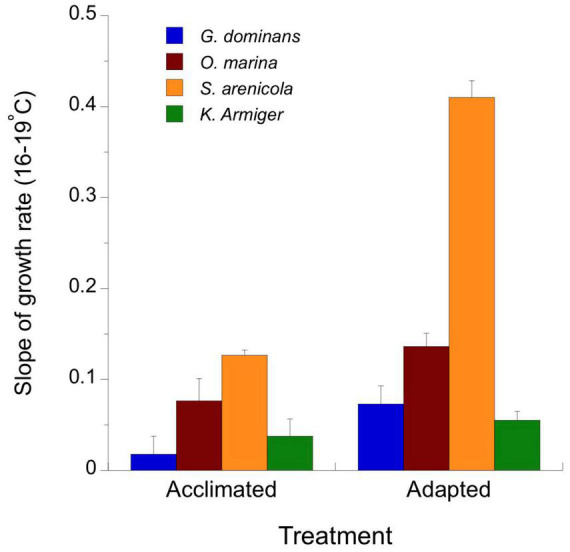
Slopes of the simple linear regression fit between growth rate and temperature for temperature-acclimated and temperature-adapted grazers. The slopes were calculated using the data showed in Figure 2 between 16 and 19°C.

**FIGURE 4:**
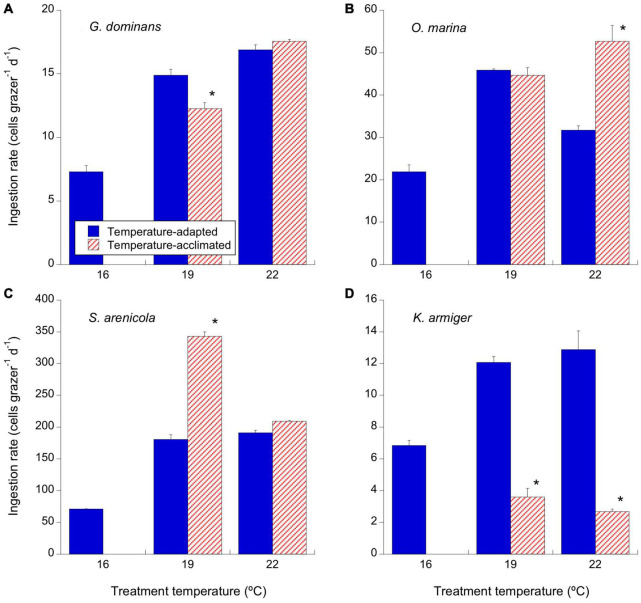
Effects of temperature on the ingestion rates (cells consumed per grazer and day) of the experimental protistan grazers. Solid blue bars correspond to the temperature-adapted organisms incubated at their standard temperature. Stripped red bars correspond to those organisms adapted at 16°C and exposed to either +3°C (19°C) or +6°C (22°C) temperature raise (2 days acclimation plus 1 day experimental incubation). Asterisks denote differences (*p* < 0.05) between acclimated and adapted treatments. **(A)** *G. dominans*, **(B)** *O. marina*, **(C)** *S. arenicola*, and **(D)** *K. armiger*. The statistics corresponding to differences between temperature treatments of adapted and acclimated organisms can be found in Table 3. The error bars are SE.

**FIGURE 5:**
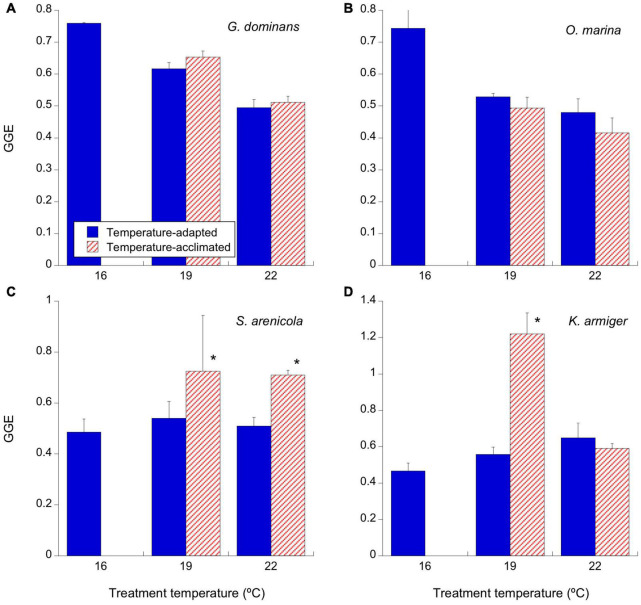
Effects of temperature on the gross growth efficiency (GGE; as the percentage of carbon produced out the one ingested) of the experimental protistan grazers (d^–1^). Given we did not have carbon data for *S. sulcatum* at 16°C, we used the values at the closest temperature (19°C). Solid blue bars correspond to the temperature-adapted organisms incubated at their standard temperature. **(A)** *G. dominans*, **(B)** *O. marina*, **(C)** *S. arenicola*, and **(D)** *K. armiger*. Stripped red bars correspond to those organisms adapted at 16°C and exposed to either +3°C (19°C) or +6°C (22°C) temperature raise (2 days acclimation plus 1-day experimental incubation). Asterisks denote differences (*p* < 0.05) between acclimated and adapted treatments. The statistics corresponding to differences between temperature treatments of adapted and acclimated organisms can be found in Table 3. The error bars are SE.

*Gyrodinium dominans* cell-based growth rates increased exponentially with temperature from 0.19 d^–1^ at 16°C to 0.48 d^–1^ at 22°C for only the acclimated strains (Figure 2A and Table 3). Adapted strains showed higher growth rates than acclimated strains at 19°C (p < 0.001), but it was the opposite at 22°C (*p* < 0.001; Figure 2A). The growth rates of *O. marina* increased from 0.66 d^–1^ at 16°C to 0.89 d^–1^ and 1.07 d^–1^ at 19°C for the acclimated and adapted organisms, respectively (Figure 2B and Table 3). At 22°C, the rates either remained the same (acclimated) or dropped to 0.8 d^–1^ in the adapted *O. marina* (Figure 2B and Table 3). There were differences in the growth rates between the acclimated and adapted *O. marina*, but they were statistically significant only at 22°C (Figure 2C, *p* < 0.05). *S. arenicola* showed a similar pattern to that of *O. marina*, with clear enhancements in growth rates at 19°C, acutely in the acclimated strains (up to 1.8 d^–1^), compared to a drop in growth rates at 22°C (Figure 2C and Table 3). Overall, the growth rates of *S. arenicola* were consistently higher for the temperature-acclimated strains than for the adapted ones (Figure 2C). The *K. armiger* thermal response was also similar to the previously described ones; however, the drop in growth rates at 22°C for the acclimated organisms was more severe than that for the adapted organisms (Figure 2D). Conversely, the growth rates for the 22°C-adapted strains of *K. armiger* were similar to those at 19°C (Table 3).

Because most species responded negatively to 22°C in both the adapted and acclimated strains, we restricted the calculation of thermal-driven enhancement of the grazer growth rates to the 16–19°C interval. Given the expected linear (not exponential) relationship between temperature (°C) and growth rates (d^–1^) in protozoans shown by Montagnes et al. (2003), in Figure 3, we present the slope of this relationship for acclimated and adapted organisms. Temperature-adapted organisms always showed higher slopes than those of their interacting counterparts; however, the differences between slopes were only statistically significant for *S. arenicola* (ANCOVA, *p* < 0.0001).

There was a consistent increase in the number of cells consumed daily by *G. dominans* and *O. marina* in their acclimated strains, from 16 to 22°C (Figures 4A,B and Table 3). This pattern was mirrored by the adapted organisms at 19°C in both species, with only significantly higher rates for the adapted vs. the acclimated strains in *G. dominans* (Figures 4A,B and Table 3). The ingestion rates dropped for the 22°C-adapted *O. marina* strain, while they remained high for *G. dominans* (Figures 4A,B and Table 3). The ciliate *S. arenicola* ingested *R. salina* at increasing rates with temperature up to 19°C (Figure 4C and Table 3); above this temperature, the rates were steady for acclimated organisms and dropped for the adapted organisms (Figure 4C and Table 3). In contrast, *K. armiger*-acclimated strains reduced their ingestion performance at increasing temperatures (Figure 4D and Table 3). This trend did not occur in the adapted strains, which showed higher rates at 19 and 22°C (Table 3).

There was an overall tendency to decrease GGE at increasing temperatures for both adapted and acclimated *G. dominans* and *O. marina* (Figures 5A,B and Table 3). However, *S. arenicola* and *K. armiger* showed slightly increasing tendencies in GGEs with increasing temperature, and those were significant only in few occasions (Figures 5C,D and Table 3). Both species responded positively to a short term increase of 3°C (acclimation experiment), and in the case of the *S. arenicola*, also to an increase of 6°C (Figures 5C,D and Table 3).

### Response to an Extreme Temperature Episode

**FIGURE 6:**
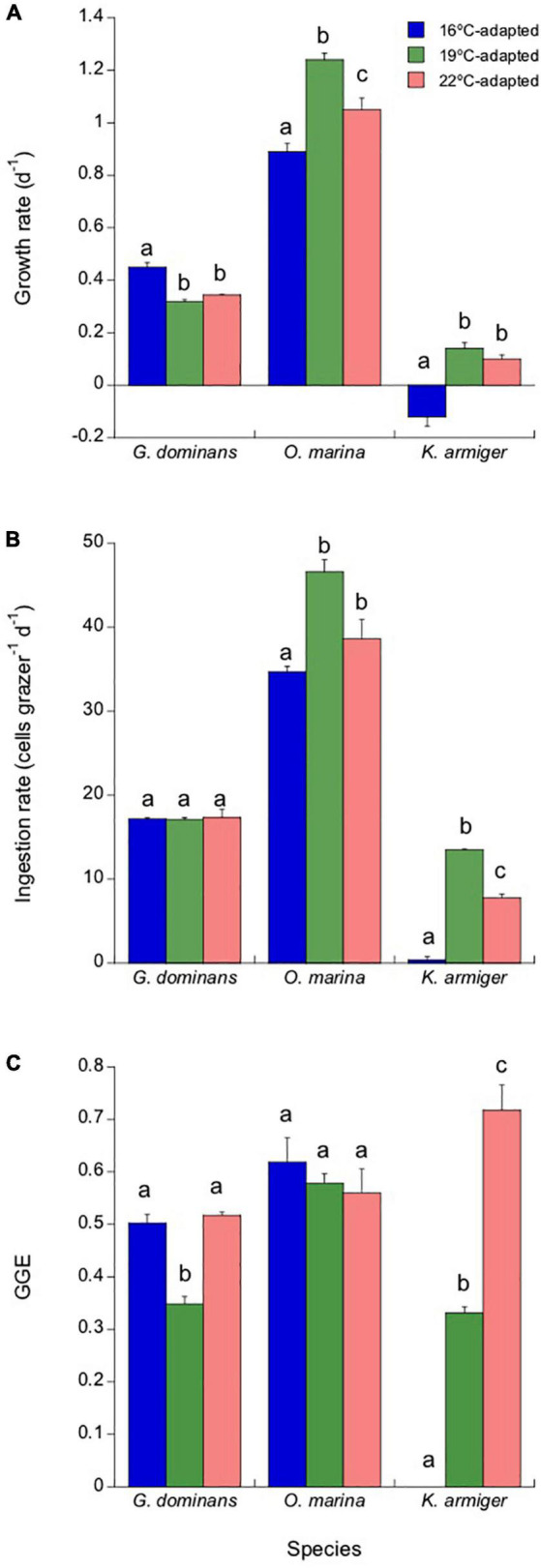
Response of the different adapted grazers (16, 19, and 22°C) to a sudden exposure to 25°C (2 days acclimation plus 1-day experimental incubation), simulating a heatwave episode. Growth rates **(A)**, ingestion rates **(B)**, and GGEs **(C)** are presented. Statistical differences (*p* < 0.05) within each species are indicated by different letters. The error bars are SE.

We present data about the response to a sudden temperature increase (Figure 6) only for *G. dominans*, *O. marina*, and *K. armiger* because the 16°C-adapted *S. arenicola* strains quickly died (within 24 h) when exposed to 25°C, and the remaining strains of this species (those adapted to higher temperatures) died after 48 h of incubation. The growth rates of *G. dominans* exposed to 25°C were very similar irrespective of the origin of the strain, although the growth rate of the strain adapted to 16°C was 26% higher than those of the other 19 and 22°C adapted ones (ANOVA and Tukey’s test, *p* < 0.001; Figure 6A). The opposite was apparent for *O. marina* adapted to 16°C, which at 25°C exhibited the lowest growth rates followed by the 22 and 19°C adapted strains (ANOVA *p* < 0.001; Tukey’s test: 16°C was different from 19°C, *p* < 0.01; 19°C different from 22°C, *p* < 0.05, and 16°C was different from 22°C, *p* < 0.05; Figure 6A). Similarly, when exposed to 25°C, the *K. armiger* 16°C-adapted strain also showed the lowest (even negative) growth rates compared to the other two strains that grew at higher and rather similar rates (ANOVA and Tukey’s test *p* < 0.01; Figure 6A). Overall, the growth rates of *K. armiger* at 25°C were low compared to the strains adapted and incubated at 19 and 22°C (Figure 2D).

The effects of incubation at 25°C on ingestion rates are presented in Figure 6B. *G. dominans* did not present any difference between the ingestion rates at 25°C, irrespective of the strain. In contrast, in *O. marina* and *K. armiger*, the strains adapted to 19°C (Tukey’s test for both *p* < 0.05) always showed the highest feeding rates, followed by those adapted to 22°C, and finally, the lowest rates were exhibited for the strains adapted to 16°C. *K. armiger* ingestion rates at 25°C were near zero for the strain adapted to 16°C (Figure 6B). The combination of ingestion and growth rates determine the values of GGE (Figure 6C). *G. dominans* grown at 19°C showed a lower GGE than that of the remaining strains of this species. All *O. marina* strains, on the other hand, showed similar GGEs at 25°C, irrespective of the temperature of adaptation. Finally, the *K. armiger* GGE was a direct function of adaptation to temperature, with the strains grown at higher temperatures showing more elevated GGEs (Figure 6C).

## Discussion

We evaluated the adaptive responses of four species of protistan grazers, including both heterotrophs and mixotrophs, to temperature. We started with several hypotheses that proved to be partially wrong. For instance, we did not find any robust evidence of lower C:N or C:P ratios resulting from temperature adaptation in either heterotrophs or mixotrophs. On the other hand, we found lower GGEs with increasing temperatures for some species, but others showed the opposite pattern. We also discovered evidence partially supporting the “hotter is better hypothesis” (Bennett, 1987), but only for a range of temperatures.

### Temperature Effects on Stoichiometry

For autotrophs, one would expect that at short time intervals (acclimation) and given the higher Q_10_ of respiration than photosynthesis predicted by the metabolic theory of ecology (López-Urrutia et al., 2006; Rose and Caron, 2007), the C:N and C:P ratios would diminish with warming. The elemental ratio decrease is explained because increased respiration over photosynthesis would make C use proportionally more favored by warming than C assimilation, reducing the overall C pool over other elements. In contrast to expectations, this pattern in elemental ratios is not commonly found for well-adapted species to temperature, particularly in terms of C:P (Yvon-Durocher et al., 2017). For heterotrophs, the stoichiometric response to temperature is not straightforward because it is impossible to separate the direct effects of temperature from those of their prey. In their meta-analysis, Woods et al. (2003) concluded that P and N contents were higher in cold vs. warm environments across various animal groups, except for prokaryotes. However, generalizations are not always corroborated by individual experiments. For instance, copepod C:P ratios decreased with temperature in nauplii and copepodites under no limitation of this element in their diets; nevertheless, when P is limiting, copepodites increased their C:P with temperature, whereas nauplii kept it low (Mathews et al., 2018). For *Daphnia* spp., the P content is negatively related to temperature, as predicted, although this relationship is species-specific and dependent on the P contents of the prey (McFeeters and Frost, 2011). For protistan grazers, unfortunately, there is an obvious need for data. Our results show that the variations in the elemental composition per unit of cell volume in adapted protistan grazers due to temperature are minor. We should consider that equal stoichiometry combined with a hypothetic reduction in volume due to temperature may nevertheless result in a lower CNP transfer (*via* mesozooplankton grazing) to higher trophic levels. Therefore, the overall effects of temperature on nutrient transfer through the food web should be considered, even if the stoichiometry of the protistan grazers does not change much.

### Does Size Matter?

Some organisms used in this study have been shown to present high body plasticity, modifying their cellular volume depending on the amount of ingested prey (Calbet et al., 2013). This fact could have masked the patterns of cell volume variations related to temperature. This does not seem to be the case because the experiments were conducted under satiating food conditions. Therefore, any change in volume during the incubations was because of a direct effect of temperature. It is surprising, then, that we did not observe a decrease in size between organisms adapted to 16°C and those adapted to 19°C. On the other hand, most of the strains adapted to 22°C, appeared smaller than those adapted at 19°C, although the differences were not significant in most of the occasions.

A decrease in the volume of autotrophs at higher temperatures, with its consequent increase in the surface/volume relationship, may favor the acquisition of dissolved inorganic nutrients. For heterotrophs, a size reduction does not seem to provide any advantage in terms of the acquisition of prey. A higher surface/volume relationship may also help obtain more oxygen, which availability is reduced by increased temperatures (Atkinson et al., 2003). Another advantage associated with the size decrease that accompanies temperature for unicellular organisms is to reduce settling rates (Atkinson, 1994; Atkinson et al., 2003). In our case, all the species considered swim, and size reduction would have only offered limited advantages. It has also been suggested that because an increased temperature will favor higher growth rates, a reduction in cell size at higher temperatures may be a response to increased population growth rates because cells that divide early will make up a more significant fraction of the total population (Atkinson et al., 2003). However, the only decrease in size we observed in our experiments (even though not significant) occurred at 22°C, and at that temperature, only *G. dominans* showed increased growth rates. In summary, our results do not clearly support the hypothesis of consistent decrease in size motivated by temperature in protozoans (Atkinson et al., 2003).

### Acclimation vs. Adaptation

Most global theories about temperature effects on plankton are mainly based on acclimated-only organisms [e.g., Rose and Caron (2007)]. Therefore, it is critical to assess whether temperature-adapted organisms show the same response than acclimated organisms. Our data showed that this is not the case for many of the variables tested. Based on growth rates, we could conclude that our organisms adapted at 16°C followed the hypothesis “hotter is better” (Bennett, 1987; Knies et al., 2009) when exposed to a moderate temperature rise (+3°C). Increases of 6°C, however, compromised the physiological performance of most of the species, except for *G. dominans*, which continued to show an enhancement in growth rates.

When considering the GGE, however, we observed that this species showed a decrease in this variable with temperature, indicating a lower efficiency of converting food into growth at high temperatures. This is not unexpected because for zooplankton, the GGE at saturated prey concentrations usually decreases with increasing temperatures (Straile, 1997; Li et al., 2011), probably due to an imbalance between physiological rates. Surprisingly, the ciliate *S. arenicola* showed higher GGEs when exposed for a short period of time to a 3 and 6°C increase. This result could suggest that this species might be favored under a future climate change scenario. However, the lack of response to temperature in the adapted strains, and the collapse of the population when exposed to 25°C, indicate that 22°C was close to its physiological thermal limit, at least for the given temporal exposure.

Finally, we want to call attention to the fact that some GGEs found in this study were at the higher limit of those reported previously (Straile, 1997). Regarding *K. armiger*, it is important to note that the very high GGE found at 19°C for the acclimated organisms may have been a combination of its autotrophic and mixotrophic metabolism. Nevertheless, for the remaining grazers, this explanation does not apply. We believe the reason for such elevated GGEs may be a consequence of the body plasticity of protozoans, in many instances a direct function of the prey ingested (Calbet et al., 2013). When considering the specific growth used to calculate GGE, we could not discern between the actual increase in biomass due to cell growth and that derived from an accumulation of prey inside the grazer’s cell. Nevertheless, even though the absolute values may be somewhat high, we feel that the comparison between treatments should be correct.

### Response to a Sudden Extreme Rise in Temperature: 25°C Experiment

The Intergovernmental Panel on Climate Change [IPCC] (2014) predicts an increase of ocean surface temperature from 0.3 to 4.8°C by the end of the twenty first century relative to 1986–2005. We have seen that many species will tolerate this increase, if gradual. However, climate change is also associated with abrupt temperature rises, known as heatwaves (Müren et al., 2005; Hayashida et al., 2020). The increase in temperature experienced by the 19 and 22°C adapted strains in the 25°C treatment was within the maximum range expected to occur in nature [up to 6.5°C, Hobday et al. (2016)], but maybe was unrealistically high for the 16°C adapted strains (+9°C). Despite this fact, however, the 16°C-adapted strains of *G. dominans* and *O. marina* showed higher growth and ingestion rates when incubated at 25°C than at 16°C (Figures 2, 4, and 6). Nevertheless, it is worth mentioning that the GGEs of the 16°C-adapted strains of all the species investigated were lower at 25°C than at 16°C, and that the maximum growth and ingestion rates of *O. marina* and *K. armiger* at 25°C were found for the 19°C-adapted strains (Figure 6). Particularly, the mixotrophic dinoflagellate seemed quite susceptible to abrupt exposure to high temperatures, dying at 25°C if adapted to 16°C, but thriving relatively well when adapted to higher temperatures. For this species, adaptation conferred clear resistance to sudden increases of temperature because the performance under 25°C was greatly improved when adapted to warmer temperatures. *S. arenicola* was the most extreme case of weak resistance to high temperature not surviving the acclimation period, and with even the 16°C adapted strain dying in less than 24 h. In summary, adaptation to warmer temperatures, even if not always fully developing the potential of the species, helps overcome the effects of short-term abrupt increases in temperature in some cases.

### Were the Strains Fully Adapted to the Experimental Temperatures?

We define acclimation as the reversible physiological changes that improve an organism’s function in the environment, whereas adaptation involves more profound genetic changes (Bennett and Lenski, 1997). In phytoplankton, previous reports suggest that a few hundred generations are enough for thermal adaptation (Listmann et al., 2016; O’Donnell et al., 2018; Aranguren-Gassis et al., 2019). However, we are not aware of similar data for protistan grazers. As these organisms also have fast generation times, one could assume a similar rule may apply. The minimum time interval the adapted strains were reared at a given temperature was 7 months, and this occurred at the highest temperature tested (22°C), meaning shorter generation times. The growth rates found in our study at 22°C ranged from 0.23 d^–1^ in *K. armiger* to 1.0 d^–1^ in *S. arenicola*, which represents less than one doubling per day for the mixotrophs and over one doubling per ciliate. It can be argued that at least *K. armiger* at 22°C may not have gone through enough generations (one hundred at most, assuming food was not always in excess) to ensure a genotypic change.

However, it is precisely in this species where we found the highest performances (i.e., growth rates) at 22°C of the adapted strains compared to those of the merely acclimated strains (Figure 2). Thus, we can conclude that even if there is likely still room to adapt to warmer temperatures, the species showed clear evidence of adaptation to high temperatures. Similarly, Huertas et al. (2011) also concluded that not all phytoplankton adapt to the temperature at the same speed. Some species are much faster than others; i.e., some algae can adapt to tolerate high temperatures after only 45 days (Padfield et al., 2016). Perhaps, the changes shown in these responses to temperature did not involve mutation but are epigenetic, and therefore, reversible. In this respect, we should be aware that despite 16°C was closer to the temperature of origin of most of our strains, after so many years in the laboratory at 19°C under a controlled environment, different from what we find in nature (no predators, minor fluctuations in the physicochemical variables, etc.), our 16°C-adapted clones could be different from the wild specimens originating the cultures. Natural selection forces in nature are different than those in the laboratory. Therefore, we cannot expect wild protists to behave the same way as our cultures. Clear evidence of this is the results of Arias et al. (2021), who found the ciliate *S. arenicola* lost its diel feeding rhythm after prolonged periods of laboratory cultivation and partially recovered it when exposed to predators chemical cues.

It can also be questioned whether our food scenario during the cultivation of the organisms could have interfered with the adaptative process. The experiments presented here were conducted at saturating food concentrations to ease the comparison between treatments. However, the thermal adaptation of the strains took place in periods of variable food conditions. Routine maintenance of cultures is designed to feed the protistan grazers every 1 or 2 weeks, allowing them consume most of the available prey. Some researchers have suggested that nutrient limitation impairs the ability of algae to adapt to lethal temperatures because of a trade-off between increased temperature tolerance and higher N requirements (Aranguren-Gassis et al., 2019). Perhaps, this situation may also be similar for heterotrophs. In our study, the range of temperatures used was below those considered lethal for the selected species, except for 25°C, which was not used to adapt any strain. We cannot discount, however, that intermittent starvation could have affected our results, explaining why our 22°C-adapted strains did not perform better than those at 19°C. Given that constant food is seldom found in nature and that food fluctuations seem to be normal (Haury et al., 1978; Mackas et al., 1985), we believe our adaptative process mimics natural communities much better than constantly fed organisms in typical experimental setups.

### Final Remarks

We can conclude that in protistan grazers, adaptation to temperature confers a selective advantage to warming within a reasonable limit (i.e., ca. +3°C), at least under food satiating conditions. Attempts to adapt to much higher temperatures (i.e., +6°C) do not confer any clear physiological advantage within the temporal framework of our experiments. Perhaps, the most remarkable exception to this was *K. armiger*, the only mixotroph that showed higher growth and grazing rates at 22°C in the adapted strains than in the acclimated strains. *S. arenicola* also showed this pattern in growth rates but not in ingestion rates. We cannot generalize with one single species that mixotrophs will thrive more than heterotrophs at higher temperatures. However, it is notable that the metabolic theory of ecology (López-Urrutia et al., 2006; Rose and Caron, 2007) predicts that heterotrophs may do better than autotrophs under a future global change scenario. Mixotrophs, showing a dual mode of nutrition, may then become more heterotrophic at higher temperatures and be favored. Interestingly, for short-term (24 h) responses to temperature, Ferreira et al. (submitted)^1^ found that in mixoplankton (including our strain of *K. armiger*), grazing was impaired at warmer temperatures, whereas photosynthesis increased. We can corroborate the results for grazing; our acclimated *K. armiger* did not respond well to warming in terms of ingestion rates. However, after proper adaptation, the ingestion rates were remarkably high (although we have no information on photosynthetic rates). Overall, this particular observation, even if not conclusive, calls for further research on the subject and a cautious interpretation of the interaction of marine protists and temperature when sufficient time to develop adaptive responses does not occur. Finally, we also want to emphasize that our experiments were conducted under excess of food and feeding on prey that were nutrient repleted. Other food scenarios would securely render different outcomes. This is particularly important because experimental assessments of temperature costs on fitness may be more evident when resources are limiting, and the other way around, food-limited organisms may have higher problems adapting to high temperatures (Aranguren-Gassis et al., 2019).

## Data Availability Statement

The raw data supporting the conclusions of this article can be found at the open repository (DigitalCSIC, http://hdl.handle.net/10261/262244).

## Author Contributions

AC wrote the manuscript and ES contributed to the manuscript revision. Both authors contributed to the conception and design of the study and approved the submitted version.

## Conflict of Interest

The authors declare that the research was conducted in the absence of any commercial or financial relationships that could be construed as a potential conflict of interest.

## Publisher’s Note

All claims expressed in this article are solely those of the authors and do not necessarily represent those of their affiliated organizations, or those of the publisher, the editors and the reviewers. Any product that may be evaluated in this article, or claim that may be made by its manufacturer, is not guaranteed or endorsed by the publisher.

## Footnotes

1 Ferreira, G. D., Grigoropoulou, A., Saiz, E., and Calbet, A. (submitted). The effect of short-term temperature exposure on critical physiological processes of mixoplankton and protozooplankton.
